# Langerhans cell histiocytosis: Presentation in a preterm neonate

**DOI:** 10.1002/cnr2.1472

**Published:** 2021-06-22

**Authors:** Ana Fadhel Alvarez, Shaily P. Patel, Maya I. Brasher, Jaclyn E. Ruggiero, Chiamaka Aneji

**Affiliations:** ^1^ Department of Pediatrics, McGovern Medical School University of Texas Health Science Center Houston Texas USA; ^2^ Department of Pediatrics, Division of Neonatology McGovern Medical School, University of Texas Health Science Center Houston Texas USA

**Keywords:** congenital rash, Langerhans‐cell histiocytosis, neonatal prematurity

## Abstract

**Background:**

Langerhans cell histiocytosis (LCH) is a rare disorder in which Langerhans cells (LC) accumulate in the skin or other organs and cause tumor formation or organ damage. Cutaneous lesions can vary widely and do not predict extent of systemic disease or prognosis.

**Case:**

We present a premature infant with skin findings, multisystem involvement, and immunohistochemical markers consistent with multisystem LCH.

**Conclusion:**

Limited data from preterm neonates with LCH suggest that prognosis is particularly poor, with even limited cutaneous disease often rapidly progressing to become fatal, although diagnosis is not always prompt. Early diagnosis and treatment may affect prognosis.

## 1 INTRODUCTION

Langerhans cell histiocytosis (LCH) is a rare disorder characterized by proliferation of Langerhans cells (LC), myeloid histiocytes originating from bone marrow that reside in the skin. LC function similarly to dendritic cells, playing a role in antigen recognition and presentation.^1^, ^2^ LCH occurs with abnormal proliferation and accumulation of immature LCs in the skin or other organs, leading to tumor formation or organ damage.^3^ Etiology is unknown but detection of specific genetic mutations, including BRAF‐V600E, support classification of LCH as neoplastic.^1^, ^4^, ^5^

LCH is primarily a pediatric disease. Incidence of neonatal or congenital LCH (NCLCH) is 1‐2/1 000 000 newborns.^6^ Disease in premature infants is rare, with only case reports published; presentation and prognosis vary significantly.^3^, ^7^, ^8^, ^9^, ^10^

Disease is classified as single‐system (SS‐LCH) or multisystem (MS‐LCH). In neonates, cutaneous disease is the most common form of SS‐LCH and accounts for about 5% of all LCH.^6^, ^11^ NCLCH most often presents as MS‐LCH, defined by involvement of two or more organ systems. Cutaneous lesions are the predominant presentation in neonates with SS‐LCH and MS‐LCH.^6^

Presence of Birbeck granules (ultrastructural cytoplasmic inclusions) is pathognomonic. Currently, diagnosis is made by immunohistochemical detection of dendritic cell markers CD1a, S100, and langerin (CD207).^11^, ^12^ Prognosis is variable and depends on multiple factors, including involvement of “risk organs” (RO; liver, spleen, and/or hematopoietic system), presence of central nervous system (CNS)‐risk lesions (involvement of skull bones), and response to therapy.^11^, ^13^

We present a preterm infant with skin findings, multisystem involvement, and immunohistochemical markers consistent with MS‐LCH. Informed parental consent was obtained and properly documented.

## 2 CASE DESCRIPTION

**FIGURE 1:**
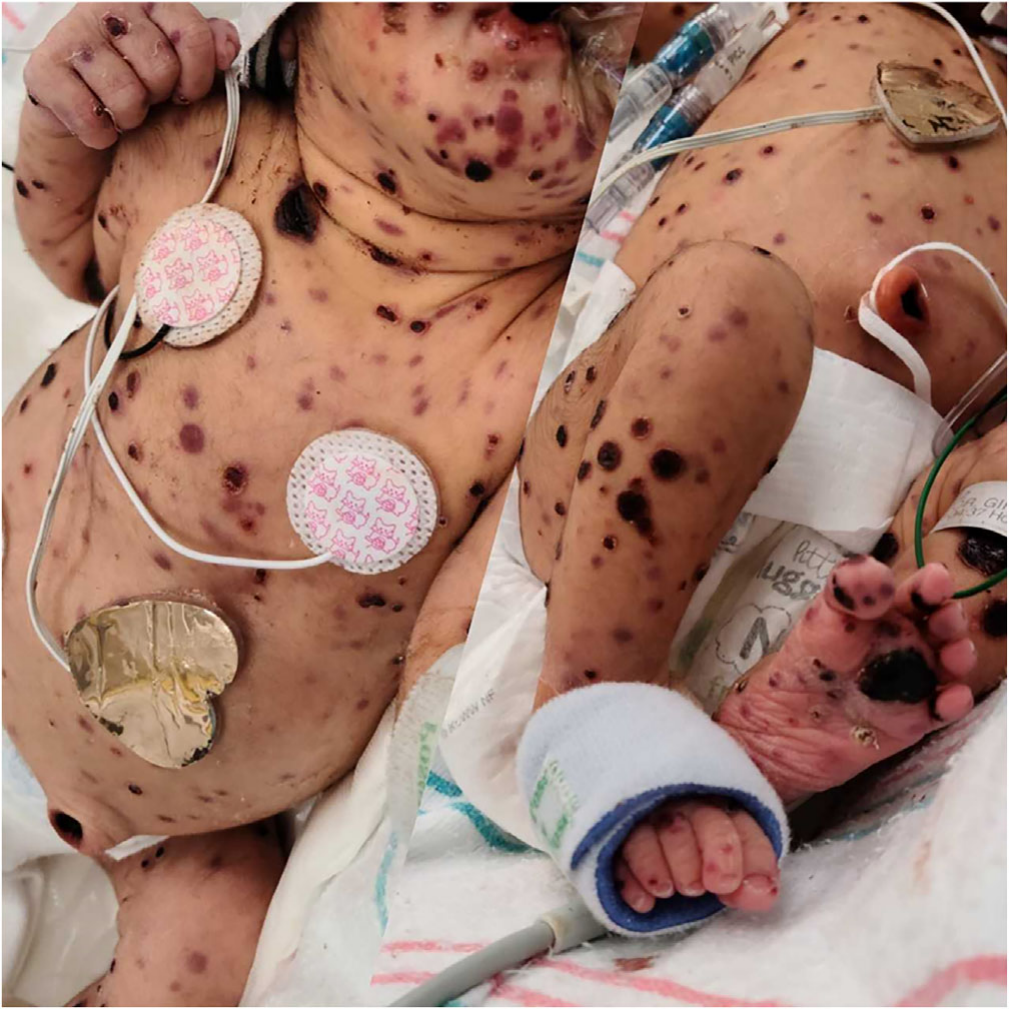
Infant's rash at birth with heterogeneous lesions. Maculopapular, pustular, and vesicular lesions were in various stages of peeling and ulceration. Lesions covered the entire body, including the palms, soles, face, and scalp

A 2.16 kg Hispanic female delivered vaginally at 33 3/7 weeks to a 26‐year‐old mother with adequate prenatal care. Pregnancy was complicated by preterm labor, prolonged rupture of membranes (24 h), and pregnancy‐induced hypertension. Prenatal laboratory results were notable for unknown group B Streptococcus status. At delivery, the neonate was limp, bradycardic, and apneic, warranting intubation. Apgar scores were 7 and 8 at 1 and 5 min of life, respectively. Physical exam revealed widespread purpuric 0.5 to 1 cm maculopapular, pustular, and vesicular lesions with peeling and ulceration (Figure 1), hepatomegaly, and diffuse lymphadenopathy.

Initial complete blood count (CBC) showed leukopenia (white blood cells [WBC] 3.8 K/ul) with 5% bands, thrombocytopenia (platelets 128 K/μl), and mild congenital anemia (hematocrit 42.6%). Peripheral smear did not identify blasts or other abnormal cells. Total and direct bilirubin at 8 h of life were 5 and 1.2 mg/dl, respectively, and continued to rise. Broad‐spectrum antibiotics, acyclovir, and ganciclovir were initiated while awaiting results of extensive infectious evaluation. TORCH testing for toxoplasmosis, rubella, cytomegalovirus (CMV), and herpes simplex virus, and for human immunodeficiency virus, varicella zoster, coxsackievirus, parvovirus, severe acute respiratory syndrome coronavirus 2, and syphilis was completed. Aspartate transaminase was mildly elevated (109 u/L) with normal alanine transaminase (33 u/L), normal uric acid (6.2 mg/dl), and elevated lactate dehydrogenase (LDH, 695 u/L).

Chest radiograph showed patchy opacities and multifocal osseous oval lucencies. Skeletal survey identified nonspecific, well‐circumscribed osteolytic lesions throughout the appendicular and axial skeleton, including cranial bones. Magnetic resonance imaging of the brain showed small intraventricular and periventricular intraparenchymal hemorrhages versus calcifications. Echocardiogram and ophthalmologic exam were unremarkable.

The patient was extubated on day of life (DOL) 3. Skin lesions were biopsied DOL 6, after infectious studies resulted negative. Newborn screens indicated severe combined immunodeficiency (primary vs. secondary), and low immunoglobulin levels prompted intravenous immunoglobulin administration to minimize risk of severe sepsis. While awaiting skin biopsy results, the patient decompensated on DOL 11 due to methicillin‐sensitive *S. aureus* bacteremia. She was re‐intubated for the remainder of her course due to hypoxic respiratory failure with severe hypercarbia requiring significant respiratory support. The infant developed severe ascites requiring drain placement and aggressive fluid replacement. Leukopenia worsened (WBC 1.4 k/μl) and she became coagulopathic, requiring transfusions of multiple blood products.

**FIGURE 2:**
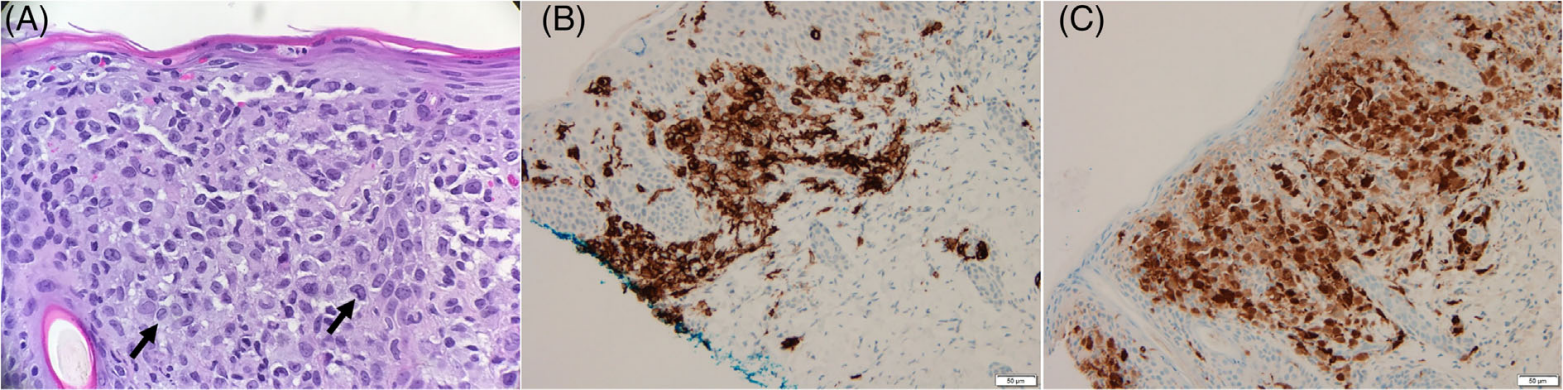
(A) H&E stained section of a skin biopsy shows Langerhans cells (arrows) characterized as infiltrating epithelioid cells with irregular folded nuclei and prominent nuclear grooves with moderate amounts of pale eosinophilic cytoplasm located in the upper dermis. Langerhans cells are highlighted by CD1a (B) and S100 (C) immunohistochemical stains

On DOL 17, skin biopsy demonstrated infiltrating epithelioid cells with immunohistochemical staining positive for S100 and CD1a, consistent with LCH (Figure 2). BRAF‐V600E staining was negative. Oncology was consulted and intravenous methylprednisolone was initiated. Bone marrow studies, positron emission tomography (PET), and chemotherapy were deferred due to clinical instability. Yeast urinary tract infection was diagnosed on DOL 25 and antifungal therapy was started. Methylprednisolone dose was increased fourfold to control disseminated disease. Despite these interventions, the patient developed multiorgan failure with severe metabolic acidosis, shock requiring vasopressors, refractory coagulopathy, and renal failure with anuria and anasarca. Given her poor prognosis, compassionate extubation was performed DOL 28. Autopsy was declined.

## 3 DISCUSSION

Diffuse congenital purpuric cutaneous lesions, or a “blueberry muffin rash,” can represent infectious, vascular, or hematologic/oncologic etiologies. Fetal dermal erythropoiesis is prolonged in infectious or hematologic etiologies in an attempt to compensate for increased blood cell production.^14^ The rash is classically associated with rubella and CMV. Congenital CMV is the most common congenital infection worldwide, with prevalence of 0.4% to 0.5% in the United States.^15^, ^16^ TORCH and other infections were considered due to our patient's “blueberry muffin rash,” premature birth, hepatosplenomegaly, and thrombocytopenia.

Cutaneous metastases from underlying neoplastic processes can present with congenital rash. Leukemia, LCH, and neuroblastoma are the three most common etiologies.^14^ Normal levels of urine vanillylmandelic acid and homovanillic acid excluded neuroblastoma. Leukemia cutis, a dermal infiltration of leukemic cells, presents as reddish‐purple nodules and occurs in 25% to 30% of congenital leukemia cases.^17^ Leukemia was unlikely with a normal peripheral smear. With LCH, cutaneous lesions can vary widely and do not predict extent of systemic involvement.^18^ Skin and radiographic findings prompted consideration of LCH in our patient.

While congenital infections are more prevalent in premature neonates, diagnoses such as LCH must be considered. Evaluation should include CBC, chemistries, liver function tests, coagulation studies, LDH, and uric acid. Consider obtaining sedimentation rate, immunoglobulins, and ferritin level. Skeletal survey should be completed, including skull views. If LCH is suspected, obtain skin biopsy, bone marrow biopsy, and PET scan for staging, with organ‐specific imaging as needed.^19^

Prognosis is affected by several factors. Studies in preterm neonates suggest that even limited cutaneous disease can rapidly progress and become fatal.^9^, ^10^ One retrospective analysis found that of 61 patients with NCLCH, 41% had SS‐LCH and 59% had MS‐LCH, with 75% of MS‐LCH cases involving RO. Compared to 6% mortality among patients with SS‐LCH and MS‐LCH without RO involvement, 54% with MS‐LCH with RO involvement died despite systemic treatment.^6^ CNS‐risk lesions predispose patients to neurodegenerative disease and predict poor outcomes regardless of treatment.^11^ BRAF‐V600E mutation is prevalent in 38% to 60% of patients with LCH and portends worse prognosis.^1^ The mutation is associated with MS‐LCH, poor response to chemotherapy, and disease relapse.^20^, ^21^ Independently, nonresponders to first‐line chemotherapy for MS‐LCH with vinblastine and high‐dose prednisone (40 mg/m^2^/day) also have poorer prognoses. Prematurity, RO involvement, earlier symptom onset, and respiratory failure are other factors associated with increased mortality.^7^, ^13^ Survivors must be monitored for long‐term sequelae. Endocrine, orthopedic, and auditory deficits are most common, while permanent neurologic, pulmonary, or hepatic involvement results in increased morbidity.^11^

Our patient presented with a “blueberry muffin rash” and complications of prematurity, with rapidly progressing multiorgan involvement. After extensive negative infectious evaluation, skin biopsy confirmed LCH and disseminated disease indicated MS‐LCH. Clinical instability precluded staging with bone marrow biopsy and PET, but prognosis was poor in setting of RO involvement. High‐dose intravenous steroids were started to control disease burden, but focus shifted to supportive care when chemotherapy could not be administered due to clinical decompensation. Ultimately, the patient succumbed to sepsis in the setting of immunosuppression from MS‐LCH.

## CONFLICT OF INTEREST

The authors have no potential conflicts of interest to disclose.

## AUTHOR CONTRIBUTIONS

*Conceptualization; investigation; methodology; visualization; writing‐original draft*, A.F.A and S.P.; *Conceptualization; investigation; methodology; supervision; validation; visualization; writing‐original draft; writing‐review & editing*, M.B.; *Conceptualization; investigation; methodology; supervision; validation; visualization; writing‐review & editing*, J.R and C.A.

## ETHICS STATEMENT

This case report has been written with institutional approval, and informed, written parental consent was obtained prior to the initiation of the case report.

## Data Availability

Data sharing is not applicable to this article as no new data were created or analyzed in this study.

## References

[1] El Demellawy D , Young JL , De Nanassy J , Chernetsova E , Nasr A . Langerhans cell histiocytosis: a comprehensive review. Pathology. 2015;47(4):294‐301. 25938350 10.1097/PAT.0000000000000256

[2] Carrera Silva EA , Nowak W , Tessone L , et al. CD207^+^ CD1a^+^ cells circulate in pediatric patients with active Langerhans cell histiocytosis. Blood. 2017;130(17):1898‐1902. 28847997 10.1182/blood-2017-05-782730

[3] Singh A , Mandal A , Singh L , Mishra S , Patel A . Delayed treatment response in a neonate with multisystem Langerhans cell histiocytosis: case report and review of literature. Sultan Qaboos Univ Med J. 2017;17(2):e225‐e228. 28690898 10.18295/squmj.2016.17.02.016 PMC5488827

[4] Hutter C , Minkov M . Insights into the pathogenesis of Langerhans cell histiocytosis: the development of targeted therapies. Immunotargets Ther. 2016;5:81‐91. 27785447 10.2147/ITT.S91058 PMC5066850

[5] Badalian‐Very G , Vergilio J , Degar BA , Rodriguez‐Galindo C , Rollins BJ . Recent advances in the understanding of Langerhans cell histiocytosis. Br J Haematol. 2011;156:163‐172. 22017623 10.1111/j.1365-2141.2011.08915.x

[6] Minkov M , Prosch H , Steiner M , et al. Langerhans cell histiocytosis in neonates. Pediatr Blood Cancer. 2005;45:802‐807. 15770639 10.1002/pbc.20362

[7] Lee CHC , Lau TK , To KF , Lam HS , Chan AWH , Ng PC . Congenital systemic Langerhans cell histiocytosis presenting as hydrops fetalis. Acta Paediatr. 2005;94(12):1843‐1847. 16431411 10.1111/j.1651-2227.2005.tb01866.x

[8] Lin H , Zhang Y , Zhang R , et al. Langerhans cell histiocytosis: a rare aetiology for fetal pleural effusion. Taiwan J Obstet Gynecol. 2020;59(5):777‐779. 32917337 10.1016/j.tjog.2020.07.029

[9] Aviner S , Ronen M , London D , Tobar A , Zangen S . Langerhans cell histiocytosis in a premature baby presenting with skin‐isolated disease: case report and literature review. Acta Paediatr. 2008;97(12):1751‐1754. 18754823 10.1111/j.1651-2227.2008.00999.x

[10] Inoue M , Tomita Y , Egawa T , Ioroi T , Kugo M , Imashuku S . A fatal case of congenital Langerhans cell histiocytosis with disseminated cutaneous lesions in a premature neonate. Case Rep Pediatr. 2016;2016:4972180. 27833773 10.1155/2016/4972180 PMC5090100

[11] Haupt R , Minkov M , Astigarraga I , et al. Langerhans cell histiocytosis (LCH): guidelines for diagnosis, clinical work‐up, and treatment for patients till the age of 18 years. Pediatr Blood Cancer. 2013;60:175‐184. 23109216 10.1002/pbc.24367 PMC4557042

[12] Satter EK , High WA . Langerhans cell histiocytosis: a review of the current recommendations of the histiocyte society. Pediatr Dermatol. 2008;25(3):291‐295. 18577030 10.1111/j.1525-1470.2008.00669.x

[13] Minkov M . Multisystem Langerhans cell histiocytosis in children: current treatment and future directions. Paediatr Drugs. 2011;13(2):75‐86. 21351807 10.2165/11538540-000000000-00000

[14] Isaacs H Jr . Cutaneous metastases in neonates: a review. Pediatr Dermatol. 2011;28(2):85‐93. 21504442 10.1111/j.1525-1470.2011.01372.x

[15] Messinger CJ , Lipsitch M , Bateman BT , et al. Association between congenital cytomegalovirus and the prevalence at birth of microcephaly in the United States. JAMA Pediatr. 2020;174(12):1159‐1167. 32926077 10.1001/jamapediatrics.2020.3009 PMC7490747

[16] Marisco C , Kimberlin D . Congenital cytomegalovirus infection: advances and challenges in diagnosis, prevention and treatment. Ital J Pediatr. 2017;43(1):38. 28416012 10.1186/s13052-017-0358-8 PMC5393008

[17] Resnik KS , Brod BB . Leukemia cutis in congenital leukemia: analysis and review of the world literature with report of an additional case. Arch Dermatol. 1993;129(10):1301‐1306. 8215495 10.1001/archderm.129.10.1301

[18] Stein SL , Paller AS , Haut PR , Mancini AJ . Langerhans cell histiocytosis presenting in the neonatal period: a retrospective case series. Arch Pediatr Adolesc Med. 2001;155:778‐783. 11434843 10.1001/archpedi.155.7.778

[19] Allen CE , Ladisch S , McClain KL . How I treat Langerhans cell histiocytosis. Blood. 2015;126(1):26‐35. 25827831 10.1182/blood-2014-12-569301 PMC4492195

[20] Hertier S , Emile JF , Barkaoui MA , et al. BRAF mutation correlates with high‐risk Langerhans cell histiocytosis and increased resistance to first‐line therapy. J Clin Oncol. 2016;34(25):3023‐3030. 27382093 10.1200/JCO.2015.65.9508 PMC5321082

[21] Ozer E , Sevinc A , Ince D , Yuzuguldu R , Olgun N . *BRAF* V600E mutation: a significant biomarker for prediction of disease relapse in pediatric Langerhans cell histiocytosis. Pediatr Dev Pathol. 2019;22(5):449‐455. 31072207 10.1177/1093526619847859

